# Antibiotic susceptibility pattern and analysis of plasmid profiles of *Pseudomonas aeruginosa* from human, animal and plant sources

**DOI:** 10.1186/s40064-016-3073-9

**Published:** 2016-08-22

**Authors:** Bamidele Tolulope Odumosu, Olabayo Ajetunmobi, Hannah Dada-Adegbola, Idowu Odutayo

**Affiliations:** 1 Department of Microbiology, Faculty of Science, University of Lagos, Akoka-Yaba, Lagos, Nigeria; 2 Department of Biosciences and Biotechnology, Babcock University, Ilisan-Remo, Nigeria; 3 Department of Medical Microbiology and Parasitology, University of Ibadan, Ibadan, Nigeria

**Keywords:** Antibiotics, *Pseudomonas aeruginosa*, Extended spectrum beta lactamase (ESBL), Plasmid

## Abstract

Multidrug resistant organisms (MDROs) constitute a major public health threat globally. Clinical isolates of *Pseudomonas aeruginosa* remains one of the most studied MDROs however there is paucity of information regarding the susceptibility of its animal and plants isolates to antipseudomonas drug in Nigeria. From a total of 252 samples consisting of plants, animals and clinical samples, 54, 24 and 22 *P. aeruginosa* were isolated from vegetables, animals and clinical sources respectively. All the isolates were identified by standard biochemical methods. Antimicrobial susceptibility testing (AST) of the 100 *P*. *aeruginosa* isolates against 7 antipseudomonal drugs was carried out by disk diffusion method, the phenotypic detection of ESBL was done by double disk synergy test (DDST) while plasmid extraction on 20 selected isolates based on their resistance to 2 or more classes of antibiotics was carried out by alkaline lysis method and analysed with Lambda DNA/*Hind* lll marker respectively. The AST results revealed highest resistance of 91 and 55 % to ceftazidime and carbenicillin respectively while highest susceptibilities of 99 % for piperacillin–tazobactam and imipenem were recorded in overall assay. Fifteen out of 100 isolates specifically (10) from vegetables, (3) clinical and (2) poultry isolates showed synergy towards the beta-lactamase inhibitor indicating production of ESBL by DDST method. Detection of plasmids was among vegetable (*n* = 4), poultry (*n* = 4), cow (*n* = 3) and clinical isolates (*n* = 1). Plasmid profile for the selected isolates revealed 6 of the strains had one plasmids each while 5 strains possessed 2–4 plasmids and 1 strain had 5 plasmids. The sizes of the plasmid range from <1 to ≥23kbp. Detection of ESBL and Plasmids among the investigated isolates is suggestive of multiple interplay of resistance mechanism among the isolates. Plants and animal isolates of *P. aeruginosa* harbouring multiple mechanisms of resistance is of concern due to the danger it poses on the public health.

## Background

Antimicrobial resistance (AMR) among bacterial species is now global public threat. AMR in bacteria is commonly associated with different resistant genes, transfer mechanisms, and antibiotic resistance reservoirs among different species. Indiscriminate use of antibiotics and prescriptions has been linked together as one of the instrumental factors leading to the emergence of multidrug resistant organisms (MDROs) in the community/hospital (Bronzwaer et al. 2002; Tarai et al. 2013). Unabated spread and proliferation of AMR genes among pathogenic bacteria has regrettably been the cause of high morbidity and mortality among critically ill patients, and has been found to contribute substantially to rising cost of healthcare due to prolong admission and expensive drugs for treatments annually (Carmeli et al. 2002; Roberts et al. 2009).

The use of antibiotics in farming and livestock management has also contributed to the spread of antibiotic resistance genes in the environment (Kümmerer 2004). In Agriculture, management of livestock and crop production often involve the use antibiotics in form of consumables that are usually excreted as biologically active metabolites in the environment. This can increase selective pressure and favours the growth of antibiotic resistant strains of bacteria (Kümmerer 2004). Studies on bacteria isolated from raw vegetables sold in different markets and eateries, that are resistant to commercially available antibiotics have been documented previously (Bhutani et al. 2015; Nipa et al. 2011).

Other studies have also focused on incidence of pet, food processing and farm animals as reservoirs for antibiotic resistant bacteria (Barber et al. 2003; Guardabassi et al. 2004; Faldynova et al. 2008). Worthy of mention are documented reports of high incidence of AMR bacteria from poultry, cattle as well other food processing animals, which are frequently consumed (Faldynova et al. 2008; Ayeni et al. 2016). It is also believed that health workers and families may serve as a conduit for the entry of resistance genes into the community and hospital environment where further spread into pathogen is possible (Molbak et al. 1999; Voss et al. 2005).

*Pseudomonas aeruginosa* is a Gram-negative, rod shaped; motile bacterium found abundant in various habitats and has been implicated in several human infections. Most *P. aeruginosa* infections are difficult to treat due to its ability to resist many structurally unrelated antibiotics, which is because of the presence of intrinsic and acquired antibiotic resistance mechanisms (Lister et al. 2009). Antimicrobial drug resistance mechanisms and its acquisition in *P. aeruginosa* are remarkable, to the favour of the pathogen. Several mechanisms of resistance to antimicrobial agents ranging from efflux pump to mobile genetic elements and hydrolysing enzymes have been described in clinical isolates of *P. aeruginosa* (Mesaros et al. 2007; Odumosu et al. 2013, 2015a, b). Notably among these mechanisms is the production of various classes of *β*-lactamases that mediates their resistance to beta-lactam drugs. Over the last decades, enzymes in this category have been detected in *P. aeruginosa*, especially extended-spectrum beta-lactamase (ESBL) such as OXA, VEB, PER, SHV and TEM types of ESBL (Odumosu et al. 2015; Mirsalehian et al. 2010).

*Pseudomonas aeruginosa* has been reported to account for up to 10 % of all human infections and is one of the important bacterial pathogens commonly isolated from various clinical and environmental samples (Rajat et al. 2012). It is also one of the major cause of diseases such as otitis, mastitis, endometritis, hemorrhagic pneumonia and urinary tract infections in both livestock and companion animals (Kidd et al. 2011; Poonsuk and Chuanchuen 2012; Salomonsen et al. 2013). However, in comparison with reported cases of hospital acquired infections, community associated infection due to *P. aeruginosa* is rarely reported (Huhulescu et al. 2011). Its ubiquity in the environment is with a high possible risk of plant-animal-human transfer of antimicrobial genes. However, due to the paucity of it’s the susceptibility from animals and plant origin to antispseudomonas drugs in Nigeria, a justification for this study was established. The objective of this study was to provide a snapshot of their resistance to antipseudomonas drug and production of ESBL. This will provide us a better understanding of how AMR are disperse in our environment and subsequently guide against it. For a good comparison, clinical isolates of *P. aeruginosa* from various samples were also included.

## Methods

Between 2014 and 2015, from 252 samples comprising of 112 clinical samples, 82 vegetables, 41 cow dungs and 17 poultry dungs, 100 consecutive and non-duplicated *P. aeruginosa* were randomly isolated of which 22 were obtained from clinical isolates, 54 from vegetables, 7 from cows and 17 from poultry. These samples were collected at different farms, hospitals at different geographical locations across south-western Nigeria states making these isolates to have different epidemiological relationships. All samples were obtained by informed consent of the patients in this study, proper ethical clearance approval was obtained from Babcock University Health Research Ethics committee NHREC/17/12/2013.

### Antimicrobial susceptibility testing

Antimicrobial susceptibility testing was performed and interpreted according to the Clinical Laboratory Standard Guidelines 2010 (CLSI 2010) on Mueller–Hinton agar (Oxoid UK) for 7 antibiotics: piperacillin–tazobactam (100/10 µg), cefepime (30 µg) imipenem (10 µg), amikacin (30 µg), ciprofloxacin (5 µg), carbenicillin (100 µg) and ceftazidime (30 µg) (OXOID UK). *P. aeruginosa* ATCC 27853 and *E. coli* ATCC 25922 were used as quality control.

### Phenotypic detection of ESBL by double-disk synergy test method (DDST)

A modified method for DDST (Jarlier et al. 1988), was performed for all the beta-lactam-resistant and intermediate resistant strains as a standard disk diffusion assay on Mueller–Hinton agar. A 0.5 Mac Farland standard suspension of the test bacteria were evenly inoculated on the Mueller–Hinton agar by gentle swabbing using sterile swabbing sticks. Disks containing 30 μg of aztreonam, ceftazidime, and cefepime, were placed 20 mm apart (centre to centre) consecutively from piperacillin/tazobactam (110 μg) and incubated for 18–24 h at 37 °C. Enhancement of the inhibition zone towards the piperacillin–tazobactam disc, indicating synergy between piperacillin–tazobactam and any one of test antibiotics, was regarded as presumptive ESBL production. *Klebsiella pneumoniae* ATCC 700603 and *Escherichia coli* ATCC 25922 were used for positive and negative controls respectively.

### Plasmid isolation and profiling

Plasmids DNA for selected ESBL positives and multidrug resistance strains totaling 20 was performed as described Kado and Liu (Kado and Liu 1981). Briefly, bacterial cultures from Mueller–Hinton broth were suspended in microcentrifuge tubes with lysis buffer, the suspension was heated for 15 min at 70 °C and mixed with an equal volume of phenol:chorophorm:isoamyl alcohol (25:24:1) for plasmid extraction. The supernatants were loaded on a 1 % agarose gel in Tris–acetate–EDTA buffer and run for an hour at 80 V. The representative plasmids were digested with *Eco*RI while Lambda DNA/*Hind*III marker was used for molecular size estimation by comparing their motilities on agarose gel with the marker. The DNA bands were visualized and photographed by using Gel Documentation system after staining with gel red and with UV transillumination.

### Statistical analysis

The Chi square test was used to compare the difference in the prevalence of isolates recovered from the various categories of samples at significant level, α = 5 %.

## Result

*Pseudomonas aeruginosa* distribution among the samples collected is as follows; poultry 100 %, vegetables 54/82 (66 %), clinical isolates 22/112 (19.6 %) and cow dungs 7/41 (17 %), The results of the antimicrobial susceptibility testing for *P. aeruginosa* shows a high-level resistance (100 %) to ceftazidime among clinical and animal isolates, while vegetable isolates showed 45 (83.3 %) resistance (Tables 1, 2, 3, 4, 5). The overall susceptibility studies show 55 and 91 % isolates were resistant to carbenicillin and ceftazidime respectively. The susceptibilities of the isolates to imipenem, piperacillin–tazobactam, cefepime, ciprofloxacin and amikacin were 99, 99, 98, 96 and 88 % respectively. Phenotypic detection of ESBL among the *P. aeruginosa* isolates was confirmed in 15 of 100. Accordingly, a total percentage of 43 positive ESBL was detected in overall, this comprised of 18 % (10/54) from vegetables, 13 % (3/22) from clinical and 12 % (2/17) from poultry isolates. ESBL was detected at the 25 mm distance synergy towards cefepime and aztreonam in all the isolates. Plasmid profiles for the selected 20 isolates are represented in Table 6. Twelve isolates harboured multiple plasmids of copies ranging from 1 to 5 and estimated size ranges of < 1 to ≥ 23 (kbp). Highest number of plasmids 4 (33.33 %) were detected from vegetables and poultry isolates, 3 (25 %) was detected in cow while only 1 (8.33 %) was detected in clinical isolates. All the isolates harbouring plasmids were resistant to carbenicillin and ceftazidime but shows complete susceptibilities to cefepime, imipenem, and piperacillin–tazobactam antibiotics. Statistical analysis shows no significant difference in the prevalence of isolate recovered from the various categories at α = 5 % significant level.

**Table 1:** Antibiotic resistance patterns of the 54 *P. aeruginosa* isolates from ready to eat vegetables in percentage distribution

Type of antibiotic	No (%) of resistant	No (%) of intermediate	No (%) of susceptible
Carbenicillin (100 µg)	34 (63.0)	6 (11.11)	14 (25.9)
Amikacin (30 µg)	1 (1.9)	4 (7.4)	49 (90.7)
Ceftazidime (30 µg)	45 (83.3)	–	9 (16.7)
Cefepime (30 µg)	–	–	54 (100)
Ciprofloxacin (5 µg)	–	–	54 (100)
Imipenem (10 µg)	–	–	54 (100)
Piperacillin–Tazobactam (110 µg)	–	–	54 (100)

**Table 2:** Antibiotic resistance patterns of the 7 *P. aeruginosa* isolates from cow

Type of antibiotic	No (%) of resistant	No (%) of intermediate	No (%) of susceptible
Carbenicillin (100 µg)	3 (42.9)	1 (14.2)	3 (42.9)
Amikacin (30 µg)	–	–	7 (100)
Ceftazidime (30 µg)	7 (100)	–	–
Cefepime (30 µg)	–	–	7 (100)
Ciprofloxacin (5 µg)	–	–	7 (100)
Imipenem (10 µg)	–	–	7 (100)
Piperacillin–Tazobactam (110 µg)	–	–	7 (100)

**Table 3:** Percentage distribution of antibiotic resistance patterns of the 17 *P. aeruginosa* isolates from poultry

Type of antibiotic	No (%) of resistant	No (%) of intermediate	No (%) of susceptible
Carbenicillin (100 µg)	14 (82.4)	–	3 (17.6)
Amikacin (30 µg)	3 (17.6)	2 (11.8)	12 (70.6)
Ceftazidime (30 µg)	17 (100)	–	–
Cefepime (30 µg)	–	–	17 (100)
Ciprofloxacin (5 µg)	–	–	17(100)
Imipenem (10 µg)	–	–	17 (100)
Piperacillin–Tazobactam (110 µg)	–	–	17 (100)

**Table 4:** Percentage distribution of antibiotic resistance patterns of 22 clinical *P. aeruginosa* isolates

Type of antibiotic	No (%) of resistant	No (%) of intermediate	No (%) of susceptible
Carbenicillin (100 µg)	4 (18.2)	5 (22.7)	13 (59.1)
Amikacin (30 µg)	2 (9.1)	–	20 (90.9)
Ceftazidime (30 µg)	22 (100)	–	–
Cefepime (30 µg)	1 (4.55)	1 (4.55)	20 (90.9)
Ciprofloxacin (5 µg)	4 (18.2)	–	18 (81.8)
Imipenem (10 µg)	1 (4.5)	–	21 (95.5)
Piperacillin–Tazobactam (110 µg)	1 (4.5)	–	21 (95.5)

**Table 5:** Antibiotic resistance patterns of the 100 *Pseudomonas aeruginosa* isolates in percentage distribution

Type of antibiotic	No (%) of resistant	No (%) of intermediate	No (%) of susceptible
Amikacin (30 µg)	6	6	88
Carbenicillin (100 µg)	55	12	33
Ceftazidime (30 µg)	91	–	9
Cefepime (30 µg)	1	1	98
Ciprofloxacin (5 µg)	4	–	96
Imipenem (10 µg)	1	–	99
Piperacillin–tazobactam (110 µg)	1	–	99

**Table 6:** Plasmid distribution among the isolates

Isolate code	No of plasmid	Plasmid profile kilobase pair (kbp)	Source of isolate	Resistance pattern
P8	5	<1, <2, 2, >2, ≥23	Poultry	CAR, CAZ, AK
S5	4	<1, ≤1, <2, ≥ 23	Vegetables	CAR, CAZ, AK
MU2	4	<1, ≤1, <2, ≥23	Vegetables	CAR, CAZ, AK
C23	2	<2, ≥23	Cow	CAR, CAZ
3L	2	<2, ≥23	Clinical	CAR, CAZ
C21	2	<2, ≥23	Cow	CAR, CAZ
S3	1	≥23	Vegetables	CAR, CAZ, AK
P14	1	≥23	Poultry	CAR, CAZ
P16	1	≥23	Poultry	CAR, CAZ, AK
C18	1	≥23	Cow	CAR, CAZ, AK
P6	1	≥23	Poultry	CAR, CAZ, AK
MU1	1	≥23	Vegetables	CAR, CAZ, AK

## Discussion

The result of the investigation in this study has revealed the various distribution of *P. aeruginosa* across the samples indicative of the pathogen ubiquity especially in the environment. A high count of *P. aeruginosa* was found in ready to eat vegetables than those isolated from animal and human sources. Similar isolation of high prevalence of *P. aeruginosa* from vegetables has been previously reported (Oluyege et al. 2015; Sabry et al. 2011). Presence of *P. aeruginosa* in animal faeces that is intentionally used as fertilizers or unintentionally in form of droppings from free-range animals in the soil suggests a possible source of contamination of the raw vegetables as well as the hands of sellers or water used in washing the food substances. Clinical isolates of *P. aeruginosa* recovered in the samples were lower in comparison to the number of samples collected. Even though *P. aeruginosa* is ubiquitous, the prevalence of its isolation among animal isolates in this study was lower compared to other general such as Enterococci and Escherichia which are commonly encountered and as previously reported from other studies (Ayeni et al. 2016; Ojo et al. 2013).

Comparing the sensitivity patterns of the isolates recovered from the three sources, it was observed that isolates from poultry showed highest number of resistance to ceftazidime and carbenicillin, the two most resisted antipseudomonas drug in this study. Amikacin resistance and intermediate susceptibility among poultry isolates were also highest (29.4 %) compared to all other sources. The observed high resistance by poultry isolates in this study suggest the possibility of selective pressure developed as a result of antibiotic use in management of poultry. It has been previously reported that food processing animals especially poultry serves as reservoirs for antibiotic resistance genes due to antibiotic use in poultry management (Diarrassouba et al. 2007; Mena et al. 2008). Such use of drugs is less common in the management of cattle and vegetable propagation because cattle rearing in Nigeria is largely carried out by local herdsman in a nomadic fashion with little or no education on the use of growth promotion in livestock business (Author’s personal observation).

In the present study, 100 % susceptibilities to ciprofloxacin, cefepime, imipenem and piperacillin/tazobactam was observed among the plants and animal *P. aeruginosa* isolates. This is in agreement with a recent study from France where a high proportion of the *P. aeruginosa* isolates from cattle and horse were susceptible to similar antibiotics as reported in this study (Marisa et al. 2015). However among the clinical isolates, there were pockets of resistance to cefepime, imipenem and piperacillin/tazobactam at 4.5 % each, while 18.2 % of the isolates showed resistance to ciprofloxacin suggesting the frequent use of these antibiotics clinically may play a significant role in its increasing resistance.

The phenotypic detection of ESBL among the isolates in this study was carried out with a modified method by including a fourth generation cephalosporin (cefepime) and tazobactam as the beta-lactamase inhibitor. Cefepime is resistant to the production of AmpC beta-lactamase commonly produced among *P*. *aeruginosa* that always mask the detection of ESBL phenotypically (Tzelepi et al. 2000). Previous studies have documented tazobactam inhibitory activity against ESBL and AmpC beta-lactamase to be almost tenfold greater than clavulanic acid (Bush et al. 1993; Phillippon et al. 2002).

**Fig. 1:**
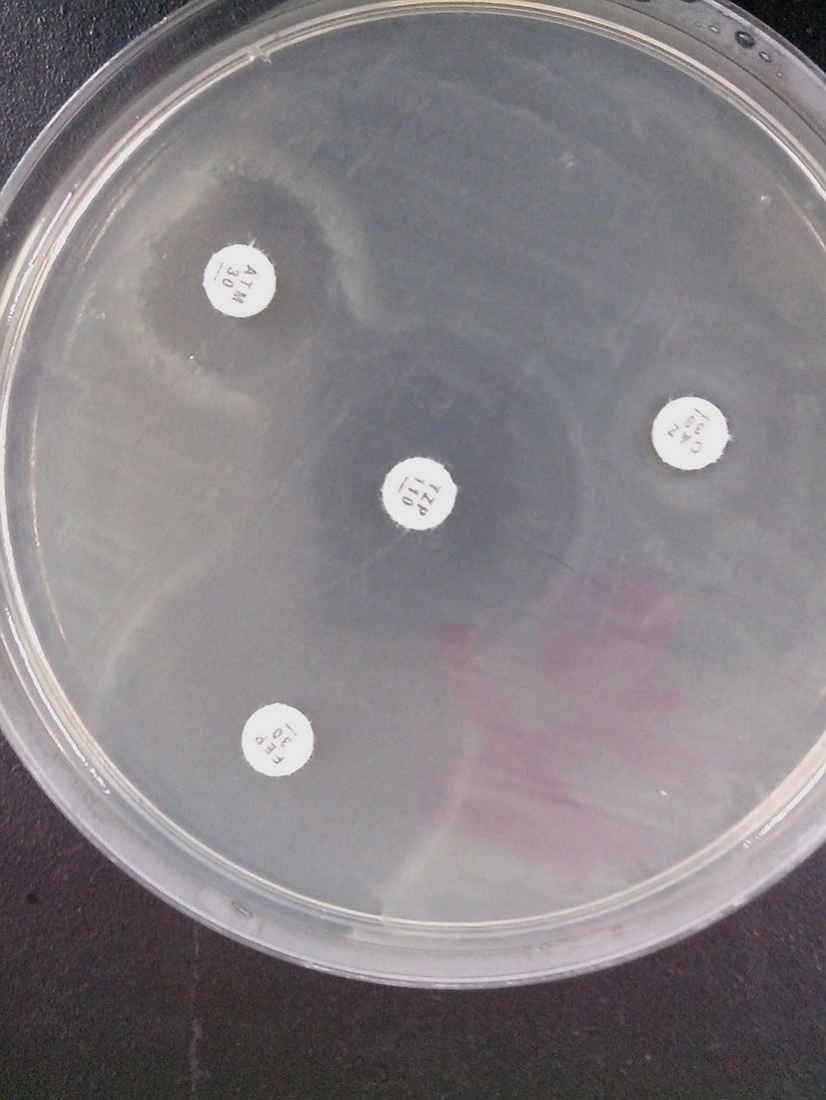
ESBL detection using DDST with piperacillin/tazobactam as beta-lactamase inhibitor

**Fig. 2:**
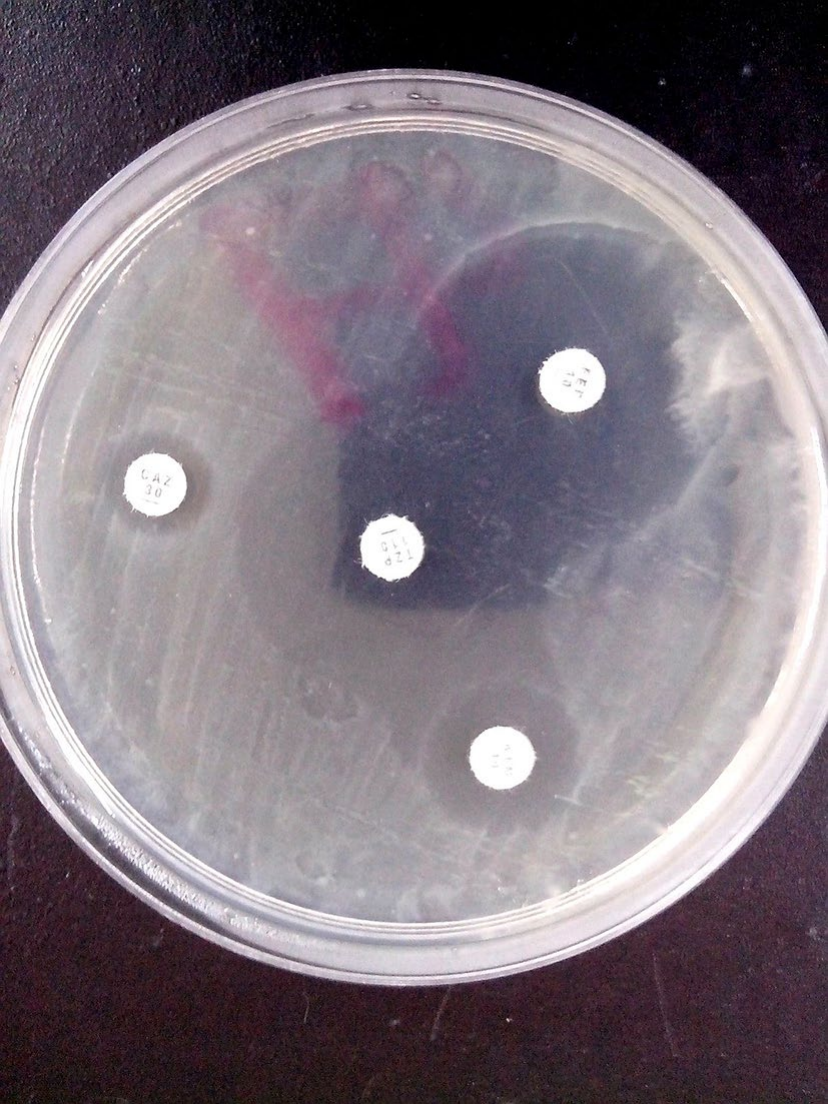
ESBL detection showing synergy towards cefepime and aztreonam antibiotics disk

Out of 100 isolates investigated for ESBL phenotypically, 15 % of the isolates were positive with synergies towards cefepime. This result is higher than observed frequency of 4.0 and 8.1 % reported from Turkey and Iran respectively (Gençer et al. 2002; Tavajjohi et al. 2011) but is in contrast with two previous Nigerian studies that reported higher frequencies of ESBL in *P. aeruginosa* (Aibinu et al. 2007; Akinjogunla et al. 2010) although percentages sometimes is a reflection of the population of the study sample hence in essence may not reflect higher or lower proportion perse (Figs. 1, 2).

In the present study, high resistance to third generation cephalosporin (ceftazidime) and carboxypenicillins (carbenicillin) both of beta-lactam class of antibiotics is worrisome. Ninety-one percent (91 %) of the *P. aeruginosa* investigated from clinical, animal and plant sources were resistant to ceftazidime in this study while 55 % were resistant to carbenicillin. According to previous studies, resistance to ceftazidime and carbenicillin by *P. aeruginosa* is mostly due to the presence of extended-spectrum beta-lactamases such as OXA types and PER that are commonly detected in *P. aeruginosa* and are usually encoded on mobile genetic elements such as plasmids and integrons (Odumosu et al. 2013, 2015; Alikhani et al. 2014). Interestingly the *P. aeruginosa* investigated in this study for the presence of plasmids all had diverse plasmids profile with copies ranging from 1 to 5 and estimated size ranges of <1 to ≥23 (kbp) (Table 6). Plasmids are mobile genetic elements and can also facilitate the dispersal of resistance genes among the bacterial population and can also serve as vehicle for other resistance mechanisms. Although the present study did not investigate the association of plasmids with the observed resistance, previous studies have shown plasmids with capacity for multiple resistance to antimicrobial agents. Incidence of these genes in animal and plants for human consumption is dangerous because of the risk of transmission of resistant strains from animal origin to humans. Cattles and poultry are frequently consume in Nigeria as part of normal diet and some people eat roasted chicken and beef without proper boiling to kill all the available pathogen. This may lead to serious public health hazard if such animal or plant sources are consumed.

## Conclusion

The present study provides a view into the antibiotic susceptibility profile of *P. aeruginosa* isolated from plants animals and humans to antipseudomonas drugs. The multiplicity of antibiotic resistance, detection of ESBL and plasmids among the isolates in this study is disturbing considering the fact that it establishes their potential abilities to the spread of antimicrobial resistance. The abundance MDROs from animal and plants in this study suggests that risk of human susceptibility to AMR bacteria from these sources is plausible. Although this study did not provide a direct evidence of their link and to what extend these sources contribute to AMR in comparison to clinical sources and hospital environments. Urgent measures are necessary to restrict the continuous abuse of antibiotics especially in livestock production. Further studies will be carried out in continuation, to fill the pending gaps in this study such as recognizing the relationship between the AMR bacteria from the animals and humans.
